# Industry-defined opportunities for advancing big data capabilities in biopharmaceutical manufacturing

**DOI:** 10.3389/fbioe.2026.1886721

**Published:** 2026-09-04

**Authors:** Roger Hart, Sandeep Kedia, Rachel Lanspa, Sarah Lichtner, Kelvin H. Lee

**Affiliations:** 1 The National Institute for Innovation in Manufacturing Biopharmaceuticals, Newark, DE, United States; 2 Nexight Group, Silver Spring, MD, United States

**Keywords:** big data, biopharmaceutical manufacturing, capability, manufacturing lifecycle, process improvement, resource-based view

## Abstract

Biopharmaceuticals are the originating products of biotechnology, an area of increased focus for strategic U.S. leadership. The discovery, development, and manufacturing of biopharmaceuticals create large amounts of data, which is then managed and preserved for business and regulatory purposes. In accordance with the resource-based view management framework, this data is a resource which, when combined with the right capabilities, is foundational to building a company’s competitive market advantage. Big Data Capabilities to acquire, analyze, curate, store, and use data for targeted purposes across the entire biopharmaceutical manufacturing lifecycle are key to realizing business value from big data collections. This paper describes these capabilities in relation to recent advances in biopharmaceutical manufacturing and the benefits these capabilities can help realize. We argue that developing shared capabilities benefits both individual businesses, who can adopt and adapt them to establish their own strategy and competitive advantage, as well as the entire industry, which advances biopharmaceutical science through shared knowledge and collaboration. This paper was derived from extensive material collected from biopharmaceutical manufacturing industry subject matter experts gathered by the Big Data Program of the National Institute for Innovation in Manufacturing Biopharmaceuticals (NIIMBL).

## 1 Introduction

Biopharmaceuticals are therapeutic products such as proteins, vaccines, and cell and gene therapies that are produced by or from living organisms using recombinant DNA (rDNA) technology and molecular biology methods (Kesik-Brodacka, 2018; Szkodny and Lee, 2022). These products pose several advantages over conventional pharmaceutical drugs: they have higher target specificity and therapy efficacy, fewer side effects and improved safety, and can be effective for patients who do not respond to conventional treatments (Kesik-Brodacka, 2018). Because biopharmaceuticals can be more effective than conventional drugs, the field has seen rapid expansion since the emergence of rDNA technology in the mid-to-late 20th century (Szkodny and Lee, 2022). The U.S. government recognizes the need for scaling up production and promoting innovation in biotechnologies like biopharmaceuticals; as these emerging technologies redefine global markets and supply chains, robust investment and policy support for biopharmaceutical innovation is essential for safeguarding U.S. technical leadership in this industry (National Security Commission on Emerging Biotechnology, 2025).

Manufacturing biopharmaceuticals involves complex processes, facilities, equipment, information systems, and networks of manufacturers and suppliers—which collectively generate large amounts of data. Historically, cell and molecular biology research has created some of the world’s largest data collections (e.g., Human Genome Project, Protein Structure Database), which now inform the discovery and design of therapeutic agents using predictive algorithms such as AlphaFold (Desai et al., 2024). Building on the successes of these earlier biology-focused data projects, the biopharmaceutical manufacturing industry can realize a similar transformation with effective use of manufacturing data.

Biopharmaceutical manufacturing companies have large amounts of data due to prior development efforts and regulatory requirements for product development and commercial manufacturing. Collecting and storing this data is costly, but using the data as a predictive asset transforms what is currently a liability into an asset. The ability to leverage these big datasets and ensure their quality and interoperability with different manufacturing equipment, systems, and vendors is important to continued growth in this industry. Manufacturers of all sizes need to be able to use their data to improve their product development, manufacturing, and supply chains *via* transfer learnings from legacy to new products, and to ultimately move more high-quality biopharmaceuticals to market.

According to the Resource-Based View (RBV) strategic management framework, organizations need specialized resources such as software, data repositories, smart equipment, and data and computer science talent to leverage big data. Organizations use these resources to develop capabilities that enhance their competitive advantage, which informs their unique business strategy (Grant, 1991). Empirical cross-industry sector RBV analyses have been conducted which show superior cost and profit-based performance of companies who successfully integrate IT resources and capabilities (Bharadwaj, 2000; Ravichandran and Lertwongsatien, 2005; Sabharwal and Miah, 2021; Teng et al., 2022). Important findings include the ability to mobilize and deploy IT-based resources in combination with other resources and capabilities to establish core competencies. The benefits were also shown to extend to cross-enterprise activities like supply chain collaboration.

Organizational resources are managerially configured by integration of individuals with specialized knowledge to create organizational capabilities which formulate and preserve knowledge. New product development, for instance, requires the integration of an extremely broad basis of knowledge, but communication constraints limit the number of individuals who can be directly involved (Grant, 1996). Empirical cross-industry sector RBV analyses similarly show business benefit from the effect of IT capabilities on cross-functional capabilities (Lin and Bush, 2010; Lu and Ramamurthy, 2011; Mikalef et al., 2018; Sabharwal and Miah, 2021). Important findings include the benefit of IT capabilities on the dynamic capabilities of the firm including innovation of new products and agile operational adjustment to respond to market or demand changes.

Empirical studies of the impact of IT capabilities within the pharmaceutical/biopharmaceutical sector are more limited and tend to focus on drug discovery or development rather than manufacturing. An empirical RBV study of a single multinational pharmaceutical company focused on the process of adoption of big data capabilities across the entire enterprise but did not examine the manufacturing lifecycle of the drugs (Grishikashvili, 2019). A separate empirical RBV study examined the impact of AI on biopharmaceutical pre-clinical drug discovery but not later stages of the product development or manufacturing (Lou and Wu, 2021). Literature reviews of the challenges in pharmaceutical manufacturing and the opportunities for digitalization have aided understanding (Arden et al., 2021; Sarkis et al., 2021). There is a gap in the literature of cross-enterprise subject matter expert advice regarding the most important big data resources and capabilities to address challenges and create benefit within the biopharmaceutical manufacturing lifecycle.

This paper expands the understanding of big data capabilities for the biopharmaceutical manufacturing lifecycle from the RBV perspectives and adds to literature examining big data capability adoption and benefits. It provides a framework for implementation of big data capabilities with considerations specific to the biopharmaceutical manufacturing industry. Our framework focuses on shared big data resources and capabilities for biopharmaceutical manufacturing, developed by manufacturers, regulators, suppliers, and academics, which can be adopted and transformed by biopharmaceutical manufacturers for their unique competitive advantages. It was informed by significant input from biopharmaceutical manufacturing industry members representing different companies and organizations. It also leverages the big data value chain structure outlined in the Horizon Europe analysis (Cavanillas et al., 2016) which conceptually maps the business context (in this case the biopharmaceutical manufacturing lifecycle) to the Data Value Chain. Due to the richness of the data collected from community experts and text limitations, the tools and offerings from the data value chain and how they enable these capabilities in biopharmaceutical manufacturing will be discussed in a forthcoming publication.

**FIGURE 1:**
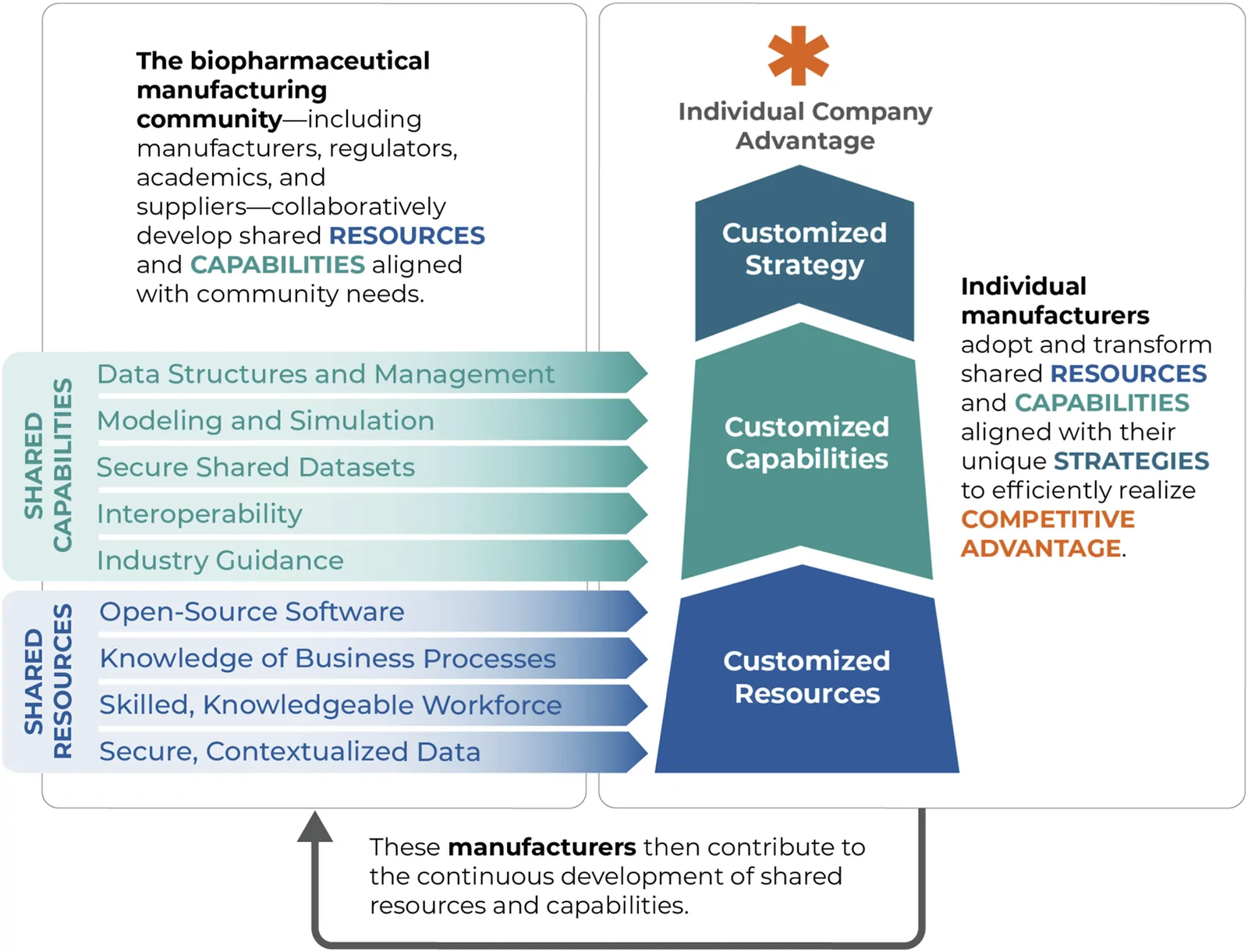
The value realization lifecycle of shared resources and capabilities for the biopharmaceutical manufacturing industry. Shared resources and capabilities are economically created collaboratively and realize benefits to the ecosystem while individual companies customize those resources and capabilities for their individual competitive advantage.

We believe the optimal path to realize full value of big data resources and capabilities involves companies collaborating to address challenges and developing resources and capabilities of shared benefit rather than pursuing similar goals separately with limited resources. Shared, openly accessible, resources and capabilities would benefit all companies and still allow them to build their own competitive advantages. Figure 1 depicts an RBV representation of how shared resources and capabilities facilitate value creation for individual companies and the broader biopharmaceutical manufacturing industry. The NIIMBL Big Data Program is actively advancing projects to mature select capabilities for its members and the larger community.

## 2 Materials and methods

NIIMBL worked with Nexight Group to convene various stakeholders from across the biopharmaceutical manufacturing industry to collect empirical evidence related to the challenges and opportunities for the use of big data in biopharmaceutical manufacturing. Through use of stakeholder engagement tools including XLeap, Slido, and Trello, NIIMBL and Nexight collected and reviewed diverse and comprehensive industry subject matter expert inputs on current industry needs and challenges, opportunities to advance big data capabilities through collaborative activities, and resulting benefits to the field of biopharmaceutical manufacturing. That information was synthesized into the comprehensive analysis presented in this paper, based on the RBV framework with influence from the European Commission’s Horizon Europe Programme for effective use of big data in the European Union (EU). Specifically, this analysis leveraged the concepts outlined in the roadmap on the usage and exploitation of big data across different sectors in the EU, especially the health sector (Cavanillas et al., 2016). This approach ensured that the resulting review and framework of this paper benefits from global efforts and best practices for leveraging big data while addressing the biopharmaceutical manufacturing sector-specific challenges in leveraging big data for improved product development processes and patient outcomes. Our paper builds on similar cross-company subject matter expert engagement for big data capability adoption accomplished in previous studies but is the first effort to do so for the biopharmaceutical manufacturing industry.

### 2.1 March 2023 workshop

In March 2023, NIIMBL hosted a 2-day, hybrid workshop—with in-person attendance at the NIIMBL headquarters in Newark, Delaware, and virtual on-line attendance—that included more than 130 biopharmaceutical manufacturing stakeholders from industry, academia, and government. The format of the workshop leveraged a professionally designed agenda and focus questions for the discussion that engaged all viewpoints through significant debate and worked toward identifying areas of consensus without driving the participants toward “groupthink” results. On the first day of the workshop, the NIIMBL-Nexight team leveraged XLeap (a browser-based brainstorming tool) to accomplish the following: 1) gather information on needed big-data-related capabilities in the biopharmaceutical manufacturing industry, and 2) collect a more objective view of priorities across diverse stakeholders through a blind voting exercise with all participants. Concurrently, the inputs and resulting priorities were curated by a small team of on-site volunteers to combine like ideas into a master list that was voted on one final time. The second day of the workshop was dedicated to further refining and prioritizing the master prioritized list with NIIMBL members only. Additional off-line work was performed after the event to categorize this extensive information by capabilities and sub-capabilities.

### 2.2 NIIMBL June 2023 National Meeting

The information captured from the workshop was then shared with approximately 30 NIIMBL members during a session at the in-person NIIMBL National Meeting in June 2023. Session participants took part in an information gathering exercise *via* Slido to answer discussion questions related to the prioritized capabilities from the workshop. The questions covered topics including the relevance of the capabilities to the products that the members work with, the potential benefits of having these capabilities, potential barriers to their implementation, and how those barriers could be overcome. Open discussion followed the Slido to build on the inputs received.

### 2.3 Synthesis and analysis

Following these two engagement opportunities with a cross-section of the biopharmaceutical manufacturing community, Nexight and NIIMBL conducted a more comprehensive analysis of all inputs from XLeap, Slido, Trello, and workshop discussions. Inputs were further categorized into capabilities and sub-capabilities and mapped in an Excel sheet to categories reflecting the applicable stages of the biopharmaceutical manufacturing lifecycle and stages in the data value chain; the results of this synthesis are included in the supplement to this publication. Further research was conducted to contextualize the capabilities and their benefits in response to current trends and drivers, as well as emerging advances and tools. The resulting analysis serves as the foundation of the NIIMBL Big Data Program strategy and a framework for how it identifies and advances priority projects; the Program is working on a subset of the capabilities or sub-capabilities outlined in the paper.

Ultimately, this strategy was informed by extensive input from the biopharmaceutical manufacturing community and directly reflects their challenges and needs. These stakeholders should feel encouraged to implement this same strategy to realize the potential of big data capabilities and maximize the impact of shared resources.

The following sections in this paper define and describe the biopharmaceutical manufacturing lifecycle stages as well as the capabilities and sub-capabilities that were identified through this synthesis.

## 3 The biopharmaceutical manufacturing lifecycle

Therapeutic product development follows a lifecycle that spans discovery/R&D through preclinical and clinical studies, regulatory approval and registration, and post-marketing surveillance. Within this lifecycle exists the biopharmaceutical manufacturing lifecycle, which includes the following iterative stages: Process Design, Technology Transfer, Facilities and Engineering, Supply Chain, Manufacturing Operations, and Quality Operations.

Each of these stages is revisited throughout the product development lifecycle as manufacturers adjust and scale their processes, add new equipment, shift suppliers, and include additional manufacturing sites. Process Design, in particular, is revisited regularly to ensure that manufacturing processes can produce the quality and quantity of product needed to address product development and market needs. Owing to the high risk of failure of potential candidates during the product development lifecycle, more costly aspects of process design (e.g., optimization, characterization) are usually delayed to later phases of clinical testing prior to submitting a marketing authorization application.

While stages are revisited regularly and manufacturers adjust their processes, facility design, supplier network, and manufacturing network, the industry faces challenges implementing more advanced and innovative approaches. The following sections define each biopharmaceutical manufacturing lifecycle stage and highlight trends, drivers, challenges, and needs at each stage.

### 3.1 Process design

Process design entails the creation and documentation of repeatable serial steps, using defined resources, to accomplish a specific outcome such as the manufacture of a specific product. Biopharmaceutical drugs are synthesized by living cells in production-scale vessels and then purified, concentrated, and formulated for use as a medicine. As cells grow, they produce many chemical entities that are considered process- or product-related impurities, which must be removed prior to concentration and formulation in excipients to support stability of the biopharmaceutical active agent as the drug substance. The resulting drug substance is later formulated, concentrated or diluted, and dispensed to specific volumes in vials and devices as the drug product used as a medicine.

Developing biopharmaceuticals is a high-risk endeavor, as these products have significant efficacy and safety challenges due to their complex biology, potential immunogenic profiles, and delayed toxicities (Youssef et al., 2025). As a result, only about 10% of pharmaceuticals make it to the commercial production stage (Sun et al., 2022). Biopharmaceuticals are more complicated than pharmaceuticals, which makes their development even riskier for manufacturers. However, when big data principles are integrated into process design, they enable the use of advanced processes (e.g., continuous bioprocessing, glycosylation profile manipulation) from the beginning of the process design that help speed product development across scales (Kornecki et al., 2019; Loebrich et al., 2019).

#### 3.1.1 Trends and drivers

In recent years, biopharmaceutical products have diversified to include many new modalities such as fully engineered proteins and polynucleotides. These new product types lack extensive data, information, and knowledge associated with prior platform products like monoclonal antibodies. Additionally, patients desire more convenience and effectiveness in their treatments and are pushing for products that can cure their condition by targeting underlying causes rather than just treating their symptoms. This desire for personalized medicine involves developing treatments based on individual patients’ unique circumstances and needs; these process/product contexts are inherently data limited. To develop individualized cell and gene therapy biopharmaceuticals, for instance, process design must account for analysis of individual molecular, physiological, environmental exposure, and behavioral profiles (Goetz and Schork, 2018). To address these problems, biomanufacturers will need to incorporate insights from genomics, proteomics, and other shared elements of biology and biochemistry to tailor processes to produce the more diverse and unique products. Example innovations include oral delivery of biomacromolecules, personal monitors, autonomous dose regulation, and release of several doses from a single administration (Baryakova et al., 2023).

#### 3.1.2 Challenges and needs

Effective process design requires a deep understanding of biological processes as well as the ability to collect data, integrate and manage complex data streams, navigate regulatory landscapes, and optimize operational workflows. Limited data collection and analysis resources can restrict the volume, variety, veracity, and velocity of data available for model training, and it may take years for algorithms to be able to run using actual production data (Steinwandter et al., 2019). The impact of high-throughput experimentation systems is hindered by a lack of corresponding high-throughput analysis, as human experimentalists are often overwhelmed by the level of data produced. Even if data collection and analysis are not resource-constrained, a lack of standardization of data types, structures, and quality inhibits companies from effectively using their own data. For example, the siloing of data across departments, systems, and business units curtails effective decision making and business operations (Abdel-Aty and Negri, 2024).

A lack of data integration across the product lifecycle deters the development of robust and accurate predictive models. Inflexible data structures hinder integration from diverse sources and instruments, which is crucial for creating a comprehensive data collection process to support advanced analytics. Unified standards are vital for effective integration of diverse data sources. Inconsistencies in user-generated content and varying standards across instruments and formats can impact data integrity and usability. Data security vulnerabilities can also expose sensitive information to potential tampering, necessitating robust security measures to maintain trust and protect data.

Issues with data collection and quality also impact a company’s ability to use and validate models for process and operational efficiency improvements. Companies need advanced models that incorporate accurate scaling and equipment capability information to ensure processes are designed to be compatible with facility constraints and that communication between equipment is effective. This is particularly true when different contract development and manufacturing organizations (CDMOs) are engaged during the lifecycle of a process. Currently, incorporating a model into control software requires additional work, contributing to higher costs. Development of platforms to make this implementation more seamless would improve the efficiency of data-driven process improvements and ensure that biopharmaceutical development can advance more effectively.

Developing novel products requires novel processes, which can introduce challenges with obtaining regulatory approvals. For example, the existing national and international legal frameworks for gene editing are racing to catch up with the technologies themselves (Kannan and Najjar, 2020). Streamlined regulatory pathways could facilitate faster validation and approval of novel process designs, which would accelerate the time-to-market for groundbreaking therapies and treatments, including gene editing, novel drug delivery systems, and advanced biologics production techniques (Kannan and Najjar, 2020).

### 3.2 Technology transfer

Technology transfer is the transfer of all the knowledge and experience necessary for creating a consistent, safe, and high-quality product (Abraham et al., 2015). Technology transfer entails the transfer of all the embodiments of a manufacturing process from one organization to another to transform and/or implement the technology for the owner’s benefit. This phase involves the systematic transfer of process procedures, equipment specifications, raw material characteristics, analytical methods, and control strategies that enable the other lifecycle stages. The underlying data, information, and knowledge from the originating team must be aggregated and contextualized to satisfy the receiving teams’ needs and align with their capabilities. The transfer is inherently transactional as the sender knows the needs, but the receiver knows the capabilities; accomplishing “facility fit” involves working through all the details and negotiating solutions to identified problems. Any identified gaps are addressed through modifications or the integration of new elements to align the receiver’s operations with the intended technology.

#### 3.2.1 Trends and drivers

As the industry seeks to maximize production efficiency and yield and reduce waste and costs, technology transfer and qualification must not impair manufacturing production capacity. Moreover, efficient transfer to contract manufacturing organizations (CMOs) for short-term supply of new products enables delays in new factory investment and better alignment with actual long-term supply requirements.

Globalization also exerts an influence on technology transfer. Expanding into international markets requires compliance with a variety of regulatory standards. Technology transfer must address different countries’ regulatory requirements, such as varying guidelines for clinical trials, manufacturing practices, and product labeling (Forum on Drug Discovery, 2013). To ensure global regulatory approval and market access, manufacturers must develop strategies to harmonize processes and documentation. Additionally, flexibility of standards and regulations is important to support continued innovation. Technology transfer processes benefit from regulations that support novel methods and technologies, such as adaptive clinical trial designs (Kaizer et al., 2023) or expedited approval pathways (U.S. Food and Drug Administration, 2024a). Flexibility in regulatory standards can ease the transition from research to commercialization, as has been demonstrated by vaccine value chains in pandemic contexts, by providing clearer guidelines and reducing bureaucratic hurdles (Medeiros et al., 2022).

More diverse product modalities, including those for personalized medicine, are influencing technology transfer as they require the transfer of complex technologies such as biomarker identification from research laboratories to clinical settings. Individualizing patient treatment is elusive owing to the variety of genes and proteins that can influence the disease (Ho et al., 2020). Technology transfer can also involve adapting the technology to different patient populations (Mathur and Sutton, 2017) and ensuring it integrates with existing treatment protocols.

#### 3.2.2 Challenges and needs

Since technology transfer primarily revolves around the analysis and dissemination of data, it is highly susceptible to challenges related to data accuracy, completeness, and integration. Effective management of these data-related challenges is essential for a smooth and successful technology transfer, ensuring that the transferred technology performs as intended. Successful technology transfer requires seamless communication and efficient information exchange between manufacturing teams, contract managers, and suppliers. It must also integrate differing IT systems and data structures while still protecting proprietary knowledge. One challenge that successful technology transfers must overcome is integration of incompatible data formats. The lack of standardized data formats between different companies creates inefficiencies and delays during technology transfer to CMOs. Incompatibility among a company’s internal data systems also hinders data transfer between an individual company’s process design and manufacturing units.

Technology transfer can also be inhibited by difficulties with tacit knowledge capture. Defining and documenting processes can be a time-consuming and challenging task; capturing the nuances that go beyond written procedures is even more difficult. This tacit knowledge is often lost when more experienced staff depart from a company, creating gaps in the transfer process. Additionally, technology transfers are further hindered by a hesitancy to share data between companies and suppliers due to business or security concerns, leading to inherently transactional and complex interactions and exchanges.

Ensuring appropriate scale-up at receiving sites is another challenge for manufacturers. Processes developed in a laboratory setting may need significant adjustments when scaled up for production. Bulk production of biopharmaceuticals uses a variety of different host systems such as bacteria, yeast, and mammalian cells (Tripathi and Shrivastava, 2018). Similarly, equipment and infrastructure differences between the sending and receiving sites may require modifications to replicate processes. As noted above, insight related to scale-up and transfer of related processes is often tacit knowledge dispersed throughout the organization.

### 3.3 Facilities and engineering

Biopharmaceutical facilities and engineering refers to the design, construction, maintenance, and improvement of industrial facilities and their constituent systems and equipment to ensure continuing, controlled, specified performance. Biopharmaceutical facilities encompass manufacturing spaces and buildings with supporting functions, the equipment these buildings house, and the human workflows essential for maintaining them in a fit-for-purpose state. Designed for decades of operation and subject to depreciation, these facilities face significant pressures, including the need to control costs within an evolving global healthcare system that demands regionalized production to meet specific needs. Furthermore, facilities must accommodate diverse products with varying drug formats and production schedules while keeping pace with competitors' output (Estapé and Chin, 2024). Fortunately, the integration of big data-informed, equipment-specific approaches can streamline processes and minimize system failures and factory downtime.

#### 3.3.1 Trends and drivers

One of the major trends impacting facilities and engineering is the continuing transition to flexible manufacturing approaches using closed processing in single-use systems. Flexible factories integrate adaptable spaces with modular plug-and-play systems and components that can be quickly changed to enable the manufacture of different therapeutic products (Bertran and Babi, 2023). Facilities are increasingly engineered to facilitate rapid transfer, scale-up, and qualification of new products while simultaneously producing existing products. This flexibility paired with accurate facility fit predictions is essential for responding to evolving market trends and minimizing downtime and waste (Yang et al., 2013). The need to maximize efficiency and yield also drives the integration of advanced manufacturing technologies, including use of automation and data analytics to enhance production processes, reduce downtime, and ensure high-quality output that meets industry standards.

Globalization also has an impact on facility design. As companies expand into global markets, facilities must be designed to meet varying regulatory requirements across jurisdictions. This includes ensuring compliance with local standards for manufacturing practices, safety, and environmental impact. A strategic approach to facility design and operation that considers these factors is essential for achieving and retaining regulatory approval for international operations. As competition for skilled talent intensifies, facilities must also provide work environments—including thoughtful design elements such as open spaces and modern amenities—that attract and retain talent and promote employee wellbeing and collaboration, ultimately contributing to operational success.

#### 3.3.2 Challenges and needs

Effective management of facilities and engineering in the biopharmaceutical manufacturing lifecycle necessitates comprehensive understanding of scientific principles and required support infrastructure, including the ability to design adaptable environments and ensure compliance with stringent regulatory requirements. A key challenge in the engineering of facilities is the complex decision-making process around facility design. When biopharmaceutical manufacturers need to grow their capacity, they face a critical decision as to whether they should outsource it to third-party manufacturers or build their own new facilities (Siganporia et al., 2014). Using a third-party facility can be less costly but may limit flexibility for the products that can be manufactured. In contrast, building a new facility allows for tailored designs but requires significant upfront investment and a lengthy construction timeline. Multi-product facilities can require significant changeover between product campaigns, and fully closed systems can enable concurrent production of several products; addressing these needs and approaches requires expansive system engineering and alignment.

Integrating modern equipment and automation technologies into existing biopharmaceutical facilities can also be difficult. Many of these facilities utilize older systems that would require costly and expansive modifications with significant downtime. The development and deployment of advanced data science capabilities and data analytics can be inhibited by outdated facilities that were not designed to integrate them into that manufacturing environment (Steinwandter et al., 2019). The structure of tax codes can also incentivize biopharmaceutical companies to retain capital equipment through full asset depreciation to capture tax benefits. Hence, manufacturers may choose incremental upgrades that extend the life of existing systems for depreciation but come at the expense of efficiency gains through innovation. Additionally, incompatible and increasingly complex communication protocols and proprietary software across diverse vendor equipment can hamper data exchange and integration, which are critical components of process management and operational effectiveness.

### 3.4 Supply chain

The biopharmaceutical supply chain encompasses all parts of the process to deliver a product or service to a customer, including sourcing, manufacturing, shipping, storage, distribution, and delivery. Natural disasters, logistics disruptions, material and component quality issues, and other unforeseen events (e.g., the COVID-19 pandemic) can cause supply chain shortages and delays. While qualifying alternative suppliers and redundancy in distribution networks can mitigate risks arising from single-point failures, these strategies can often be cost-prohibitive in the current market. Fortunately, advancements in AI, blockchain, and data analytics are empowering companies to gain real-time visibility into their network and predict future challenges with supply and distribution. This value has already been demonstrated in case studies involving premier pharmaceutical companies such as Pfizer, Amgen, GlaxoSmithKline, Merck, and Roche (Guo, 2023).

#### 3.4.1 Trends and drivers

Downward cost pressures and shrinking product margins are driving the need for more cost-effective and streamlined supply chain operations, ultimately increasing the extent to which supply chains are vertically integrated. To maintain profitability, biopharma companies may focus on optimizing logistics, negotiating better terms with suppliers, and reducing waste (Ogbuagu et al., 2022). In particular, cell and gene therapies create unique demands for highly tailored supply chains to ensure the precise formulation and delivery of treatments within narrow timeframes. This increased complexity in logistics can require advanced tracking systems and individualized inventory management (Waldman and Terzic, 2014).

Stricter labeling and reporting requirements also necessitate more detailed documentation and tracking throughout the supply chain, which can increase administrative burdens and require enhanced data management systems to ensure compliance. To fulfill compliance requirements for advanced therapy drugs, manufacturers have to create product labels that are updated at key intervals of the cell collection and manufacturing process in real time (Suchet, 2020). There are also diverse, and often stringent quality standards introduced by globalization**,** which impact different regions of the biopharmaceutical supply chain. Companies must navigate a complex landscape of regulations, leading to increased operational costs and necessitating robust quality control and risk management systems. At both the national and international level, manufacturers would benefit from more flexible regulatory standards, which could enable supply chains to be more dynamic by allowing manufacturers to more quickly qualify suppliers or facilities in response to supply disruptions or changes in demand.

#### 3.4.2 Challenges and needs

Effective supply chain management in biomanufacturing relies on clear communication and seamless data exchange between manufacturers and suppliers. It requires overcoming issues such as incompatible IT systems, data structure discrepancies, and concerns over protecting proprietary information. Among the major challenges impacting supply chain management is the limited visibility within supply chains. Traditional methods fall short for predicting and monitoring the condition of raw materials, consumables, and finished products throughout the supply chain. This lack of visibility interferes with management and response to supply chain variables, making it challenging to ensure product integrity and optimize supply chain performance. Limited visibility into suppliers’ supply chains also impedes environmental impact and sustainability assessments and purposeful management of the environmental impact of the supply chain.

The biopharmaceutical industry also faces significant challenges due to inconsistent data formats and the absence of secure, standardized platforms for sharing information among manufacturers and suppliers. These data disparities hinder effective collaboration and obstruct seamless integration across the supply chain. Inefficiencies in inventory management systems are another significant challenge for supply chain management. Difficulties in integrating real-time process data with inventory management systems hamper accurate forecasting and optimal inventory control. This lack of synchronization leads to inefficiencies in inventory management, such as overstocking or disruptions of supply chain stability. Effective integration is crucial for ensuring inventory levels align with production and demand needs.

### 3.5 Manufacturing operations

Biopharmaceutical manufacturing operations include the planning and execution of activities involved in the production of products by transforming or combining materials using equipment in accordance with procedures. They entail the use of living organisms, biological processes, and engineered equipment in nearly ideal environments for cells to grow, divide, and be transformed into safe and effective biopharmaceutical products (Rathore et al., 2021). Traditionally, these operations took place in large factories with stationary equipment, fixed piping, transfer panels, and stainless-steel tanks. In contrast, contemporary factories are smaller, utilize movable equipment connected by flexible hoses, and employ disposable single-use systems (Shukla and Gottschalk, 2013). This evolution not only mitigates the risk of cross-contamination but also accelerates production, paving the way for dynamic manufacturing environments.

As manufacturers embrace modern design, they increasingly adopt automated and integrated modular systems to conduct sequential or continuous biopharmaceutical process unit operations. Such systems also integrate process analytical technologies, modeling, and control strategies (Chopda et al., 2022). This shift reduces costs and minimizes the number of manufacturing operators required. The flexibility of these systems allows for greater connection combinations, which both enhances operational versatility and increases the number of modes of failure. Fortunately, big data capabilities can be used to minimize deviations within these modes and automate aspects of validation.

#### 3.5.1 Trends and drivers

The adoption of Industry 4.0, or Pharma 4.0 in pharmaceuticals, is a substantial manufacturing revolution promoting the use of artificial intelligence and advanced data science tools and methods to develop lean, risk-based approaches for pharmaceutical and biopharmaceutical development (Soldatos, 2024). To address financial constraints and downward cost pressure amid quality requirements, biomanufacturers have adopted advanced technologies that streamline production and reduce costs. For instance, companies use single-use bioreactors rather than stainless steel to reduce cleaning time and risks of losing large quantities of product due to contamination (Guldager, 2010). The influence of lean manufacturing has also served to reshape manufacturing operations by introducing the continuous pursuit of efficiency and the elimination of waste. Lean manufacturing emphasizes streamlining every aspect of the process, from reducing excess inventory and minimizing downtime to optimizing workflows and cutting unnecessary costs (Guilland and Stoll, 2009). Continuous manufacturing systems help enable these advanced technologies and opportunities for streamlining processes. These include the use of closed loop control systems, which improves performance for linear processes but requires modification for non-linear processes (Rathore et al., 2021).

At the same time, regulations have become more stringent around labeling and reporting requirements. Manufacturers must provide more detailed information on product labels, including comprehensive ingredient lists and usage instructions. These requirements add layers of complexity to production and packaging for different languages and regulated markets and the need for additional resources for data management and audits.

Unlike traditional pharmaceutical manufacturing, which relies on standardized production of large batches, manufacturing of some biopharmaceuticals involves production of small, patient-specific batches. This shift has led to the adoption of modular and adaptive production systems that can quickly adjust to produce different product specifications (Siiskonen et al., 2020). As more innovative therapies are developed, manufacturers are also seeing a need for enhanced safety and efficacy measures, which have an impact on operational processes. For example, viral vectors have matured to deliver nucleic acids to patients to induce immunity or correct aberrant *in vivo* expression involved in disease. These modalities tend to be potent, requiring strict attention to containment and monitoring during manufacturing. Biopharmaceutical manufacturers must strictly adhere to Good Manufacturing Practices (GMP) to minimize risks and ensure consistency.

Another factor impacting manufacturing operations is increased competition for workforce talent. To attract and retain skilled employees, manufacturers are investing in employee training and development programs, including data science, and offering competitive compensation packages (Moutray, 2022). Additionally, companies leverage automation and advanced technologies to reduce dependency on manual labor.

#### 3.5.2 Challenges and needs

Biopharmaceutical manufacturing operations require the ability to produce a range of products efficiently using adaptable spaces and processes. To do so, manufacturers must reconfigure equipment and automate processes to meet varying product specifications while ensuring high-quality outcomes. However, a lack of systems, such as advanced sensors, for capturing and analyzing real-time process data leads to incomplete awareness of the biomanufacturing process and impedes the ability to use data to monitor and optimize operations. In addition, data formats across different systems are often incompatible. The lack of standard formats and platforms leads to fragmented data distributed across different systems and hinders data integration for holistic analysis. This disjointed approach limits the potential for comprehensive monitoring, analysis, and streamlined operations.

Manufacturers are also burdened with the continuing need for resource-intensive manual interventions. Biopharmaceutical manufacturing can require manual data entry and analysis as well as manual interactions with process controls, all of which are resource-intensive and susceptible to human error. This approach is time-consuming and diverts personnel from more strategic activities. Issues with inadequate documentation and communication of manufacturing process details pose another challenge for manufacturers, resulting in knowledge transfer gaps. These gaps become problematic when adapting production or modifying processes and increase the risk of errors and delays. Continuous manufacturing systems can reduce the need for manual interventions and thus reduce the risk of errors, but the effort to implement them for biologics production is significant, and thus has not been widely adopted (Chopda et al., 2022).

### 3.6 Quality operations

Biopharmaceutical quality control comprises the systematic approaches used to ensure delivery of products that meet predefined quality specifications. It is a critical function that relies on setting standards, monitoring processes and products, analyzing deviations, and implementing corrective actions. This stage of the manufacturing lifecycle ensures that all operations comply with GMP to maintain manufacturing licenses and enable release of drug substance and combination products for distribution to patients.

Automation has become essential for quality control and assurance because it can increase workflow speed, minimize variability, and ensure high levels of precision. Automated systems can, for instance, inspect a much larger set of samples in a shorter timeframe than if they were inspected manually (Hofer et al., 2020). Automatic data collection also enables transfer learning in machine learning models, where generic parts of the model are adjusted based on learnings from the specific system’s data; this serves as a bridge for hybrid modeling approaches to generalize knowledge across the manufacturing process informed by a target product quality profile (Sokolov, 2020). In short, big data capabilities allow for faster, more accurate assessments of product quality, improving manufacturing efficiency, reducing product waste, and ensuring patient safety.

#### 3.6.1 Trends and drivers

Biopharmaceutical manufacturing companies must have robust quality operations for accelerated decision-making in response to new information and change. Big data capabilities that enable faster problem resolution and timely updates to quality control processes are needed to ensure products remain competitive and compliant. In particular, emerging biopharmaceutical modalities, including gene and cell therapy, are driving demand for enhanced quality control tools. These modalities are complex to test analytically and have shorter expiration times compared with conventional pharmaceuticals. More complex, and rapid, multi-attribute testing, analysis, and recording are needed to assure quality and enable release and distribution to patients prior to product expiration.

Downward cost pressures are also exerting an influence on quality operations. Quality is a necessary but significant element of product costs, which must be managed to ensure product competitiveness and market share. Manufacturers must adopt cost-effective quality control measures and find innovative ways to maintain high-quality outputs while managing expenses (ICH Q13, 2022; Niazi, 2025). Regulatory pressures such as increased labeling and reporting requirements are also driving companies toward developing more comprehensive systems for accurate documentation, tracking, reporting of product information, and making product safety profiles (Lucas et al., 2022). At the same time, quality operations must be robust and flexible to handle varying regulatory requirements while ensuring consistent product quality worldwide (Global and Regional Regulatory Harmonization Initiatives, 2024). This often requires development of harmonized quality systems that can be adapted to local regulations to ensure compliance and facilitate efficient international operations.

#### 3.6.2 Challenges and needs

Ensuring robust quality operations is essential for maintaining regulatory compliance and delivering high-quality biopharmaceutical products. Quality operations require stringent monitoring and control of production processes, integration of diverse data sources, management of quality deviations, and coordination across operational stages. One of the most significant challenges facing quality operations is the limited ability to conduct real-time monitoring of critical quality attributes (CQAs). This limitation impedes timely interventions, as traditional reliance on post-run analysis delays the release of final product and the detection and correction of quality issues—the net effect being higher product inventories and wasted shelf life. Quality-by-design approaches to product development, which use CQAs as critical process parameters to inform advanced modeling that helps predict quality outcomes, require large datasets to ensure accuracy, leading to the need for digitalization across their production facilities (Walsh et al., 2022).

Additionally, manual deviation management—the process of manually tracking and investigating deviations—is resource-intensive and slow. This challenge is exacerbated by difficulties in accessing and analyzing comprehensive datasets, which are crucial to accurately analyzing deviation events to identify the root cause and corrective action. This laborious approach prevents timely resolution of quality deviations and increases the cost and duration of quarantined products. Similarly, organizations face challenges with incomplete process understanding due to limited and incompatible data. Fragmented data and limited sensor coverage hinder the real-time comprehensive understanding of bioprocesses, which leads to a reactive approach to managing quality, where issues are addressed after they arise, rather than being anticipated and prevented.

Variability in the quality of supplied materials introduces challenges to maintaining consistent product quality. The need for extensive supplier oversight and delayed feedback mechanisms creates bottlenecks that hinder quality management and process efficiency. In addition, issues with transferring and aligning process knowledge and data between sending and receiving sites during the technology transfer process can disrupt product quality consistency. This is particularly true with transfers to CMOs where data transmission is governed by contractual agreements. Challenges in integrating knowledge into the quality operations of the receiving site can lead to discrepancies in quality control. Manufacturers also face difficulty with incomplete automation and poor interoperability between equipment from different vendors, which disrupts the flow of data and hinders effective process monitoring. These integration gaps create barriers to achieving comprehensive quality control and maintaining consistent process performance.

### 3.7 Benefits across the biopharmaceutical manufacturing lifecycle

Big data is revolutionizing biomanufacturing by significantly enhancing operational efficiency and regulatory compliance as data matures to information and then to knowledge throughout the product lifecycle. By utilizing standardized data formats, companies can create prior-knowledge assessments, conduct more informed root cause analysis to identify effective corrective actions, and efficiently produce high-quality regulatory filings to reduce delays in approvals. This streamlined approach is complemented by real-time data insights linked to clinical outcomes, which empower organizations to make informed decisions regarding product specifications and process optimization.

Moreover, aggregating data across the development lifecycle enables the creation of predictive models that not only optimize processes but also minimize risks. The integration of process analytical technology (PAT) further enriches operational insights and enables advanced process control strategies, allowing for earlier and continuous monitoring and proactive adjustments that help maintain product quality (Egaña Tomic, 2021). Growing data collections and real-time monitoring over the lifecycle enables creation of digital twins, which can support use of augmented reality (AR) and virtual reality (VR) for operator training and support. Such tools speed skill development, while simultaneously reducing the risk of errors during actual manufacturing. Automating data collection and analysis minimizes human error, ensuring greater consistency in product quality.

Big data also enhances knowledge management and reuse, which increases efficiency by revealing and maturing platform approaches that can be used across product portfolios. Big data approaches enable transformation of tacit knowledge to explicit knowledge, thereby increasing company resilience to the loss of key subject matter experts and changes in the business and regulatory environment.

## 4 Big data capabilities

A business capability is a “particular ability or capacity that a business may possess or exchange to achieve a specific purpose or outcome” (Homann, 2006). Capabilities can be further split into more granular business capabilities for purposes of planning and detailed business or IT mapping; this deconstruction provides a sense of how capabilities fit in the overall business. Developing business capabilities requires extensive investment in staffing, training, and compensation, among other areas. The result of these investments leads to capabilities being highly valuable, but intangible, assets that can be reused and reconfigured. While initial investments into developing these capabilities may be significant, returns on those investments will be available to the company for many years. Ultimately, business capabilities drive extensive value for organizations, forming the foundation for their strategy and primary sources of profit (Grant, 1991).

**FIGURE 2:**
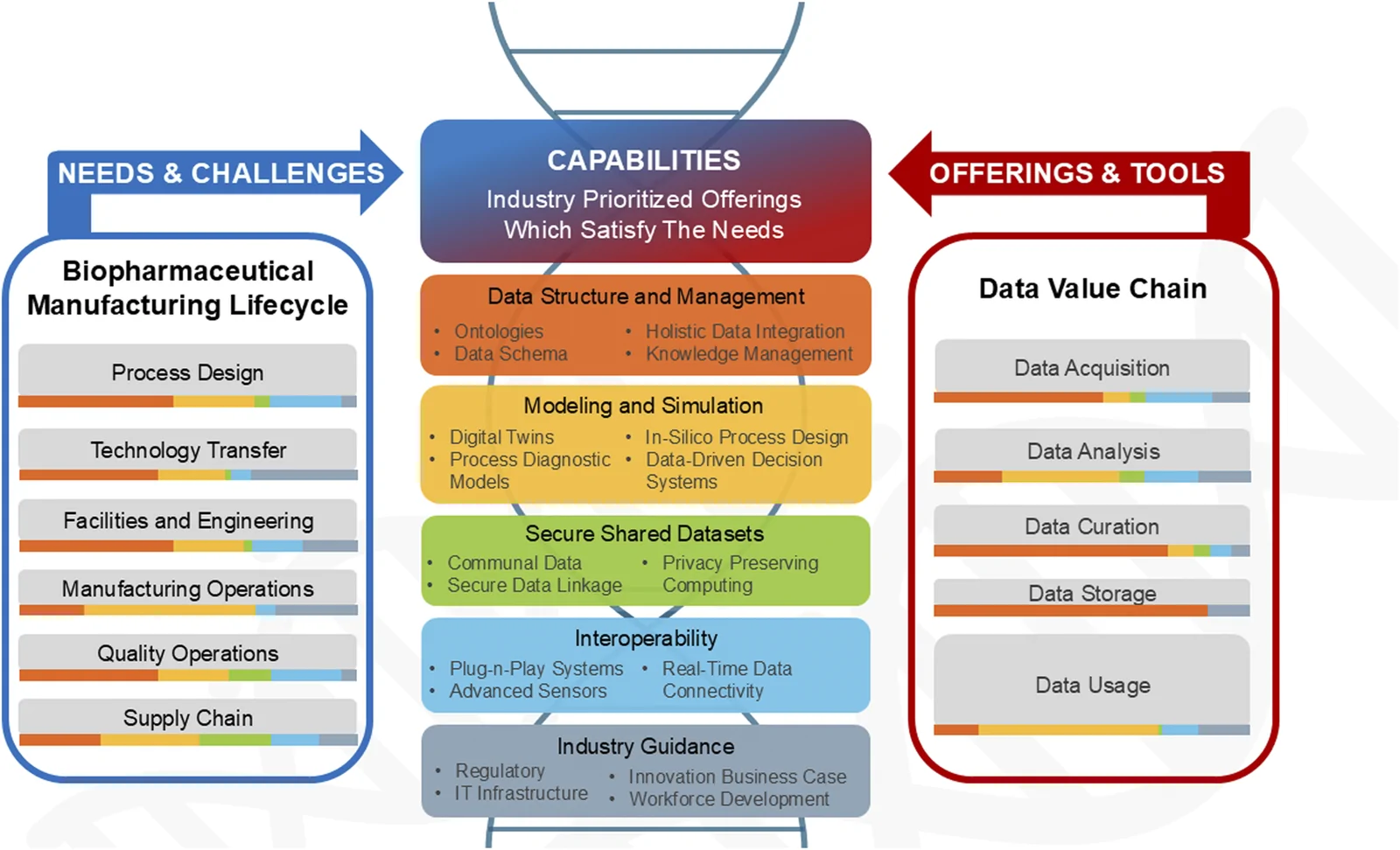
Overview of biopharmaceutical manufacturing industry-led big data capability framework. The colored bottom bars in the biopharmaceutical manufacturing lifecycle on the left and the Data Value Chain on the right correspond to the colors of the capabilities in the middle. The size of these bars is based on the number of votes received during workshop facilitated sessions to define this strategy (see Materials and Methods).

This section discusses five overarching big data capabilities: data structure and management, modeling and simulation, secure shared datasets, interoperability, and industry guidance. Each overarching capability is composed of several specific capabilities, which we call “sub-capabilities.” Synergies across capabilities are described within each capability description as well as in a table at the end of this section. These capabilities are informed by the challenges and needs of the biopharmaceutical manufacturing lifecycle and by the tools and advances from the data value chain. A visual representation of this relationship is shown in Figure 2 below.

### 4.1 Data structure and management

The Data Structure and Management capability is the method of organizing and storing data within computer systems in a structured format to enable its future access, retrieval, and use. Collectively, these computer systems are diverse and composed of many different components, each containing diverse time-spanning data created and used by many different actors across the biopharmaceutical manufacturing lifecycle. Hence, an enterprise must utilize data structures and management approaches that can be deployed in the diverse collective and systematically evolve with time. Proper data structure and data management practices are the foundation on which modeling, shared datasets, and interoperability are built, and are the key to acceptable product registration submissions. However, because this capability requires a foundational shift in data management practices, it can be difficult to implement for companies reliant on legacy systems and large sets of unorganized historical data. Pharmaceutical companies are often reluctant to completely redesign their data integration systems to incorporate big data because doing so is time- and cost-prohibitive (Grishikashvili, 2019). Significant manual effort and subject matter expert oversight are also still needed for data transfers and for equipment and product tracking within and across manufacturing sites. For these reasons, the return on investment for implementing the data management capability can be slow to fully realize.

#### 4.1.1 Ontologies

An ontology is a formal semantic model that represents a set of concepts and their relationships within a common reference model (Tolk, 2018; Wilson et al., 2024). Ontologies define standard vocabulary and relationships that enable semantic interoperability (i.e., the meaning of data is consistent) across different systems. This capability is a critical component of interoperability and is becoming a requirement for capabilities such as digital twins (Ferko et al., 2023) and operation of technologies in the Industrial Internet of Things (IIoT) (Acharya et al., 2024). Ontologies can be used to develop standard big data terminologies (such as standardized metadata), maps and knowledge graphs, contextualized datasets, management platforms, data transformation frameworks, and knowledge bases. Canonical ontologies can be used to support intra- and inter-company data transfers.

#### 4.1.2 Data schema

A data schema is a type of formal conceptual model that standardizes data content, structure, format, processes, relationships, and constraints in a way that machines can understand (Wang et al., 2009). In biopharmaceutical manufacturing, a well-designed data schema streamlines the automated extraction and integration of data from various sources, in alignment with an ontology, to facilitate efficient reporting and analysis across the manufacturing lifecycle. It encompasses standardized data formats, reference data frameworks, and common data specifications, which collectively enhance data consistency and interoperability among different manufacturing operations and systems. Standardized data schemas enable digital interchangeability of comparable instruments within a network and across lifecycles.

#### 4.1.3 Holistic data integration

Holistic data integration is an approach for data management in and among diverse sources that emphasizes interconnectivity, accessibility, and interoperability to create a comprehensive unified view of a system or process. This approach organizes and stores data across non-GMP, development, and commercial scales in a manner to enhance accessibility and knowledge transfer across the manufacturing and product lifecycle. For example, holistic integration of supply chain data allows companies to effectively oversee relationships with suppliers, customers, and logistics providers and identify alternate suppliers and interchangeable products when needed. Standard procedures for aggregating data from equipment, sensors, and integrated unit operations optimize resource utilization, enhance process knowledge, support real-time analytics, and enable adaptive control.

#### 4.1.4 Knowledge management

Knowledge management is a business process for creating, storing, using, and sharing knowledge and information within an organization. Conceptually, information results from analysis of collections of data to create a view of a historical or possible event; knowledge results from understanding and internalizing collections of information in the human mind (Liew, 2007). In biopharmaceutical manufacturing, effective knowledge management is crucial for mapping explicit information and knowledge and capturing the network sources of tacit knowledge (i.e., the know-how and practical knowledge of production tasks that is acquired by and stored within individual workers) (Grant, 1996) resulting from advanced data analysis and modeling. Establishing a robust knowledge management framework allows companies to develop highly reusable platforms and technical approaches based on the insights derived from various data inputs—such as time-series, batch, and analytical data.

### 4.2 Modeling and simulation

The Modeling and Simulation capability involves the creation and use of virtual representations of real-world systems using software and hardware to emulate and evaluate complex systems. Models are abstractions of systems constructed to facilitate simulations, which are a form of scientific experimentation and assessment (Menner, 1995). Modeling and simulations are well suited for solving problems within complex systems, like those found in biopharmaceutical manufacturing, and situations where real-world experimentation is difficult. Hybrid modeling approaches combine data-driven (statistical) and knowledge-based (mechanistic) approaches to simulate final product quality and enable experts to focus on the areas in which the most improvement and optimization is needed to ensure optimal product quality (Sokolov, 2020). Implementing this capability depends on whether organizations can access and adequately use real-time data to inform predictions and develop models, as well as the ability to gather and use additional data to help create more generalizable models in the future (Shanley, 2019). Additionally, there is limited regulatory guidance on best models/modeling tools for a given unit operation in biopharmaceutical manufacturing, as well as a lack of standard, open-source software and experimental datasets to validate models (Babi et al., 2022); this creates uncertainty around regulatory expectations for model development, which can impede the implementation of this capability. The lack of large, anonymized datasets is also a significant impediment to model development due to the reluctance to share data and potentially expose proprietary company information (Wittkopp et al., 2024).

#### 4.2.1 Digital twins

A digital twin is a virtual replica of a real-world, time-evolving object, process, or system that relies on real-time data to simulate and predict real-world behavior and offer feedback on its performance (Acharya et al., 2024). Digital twins can also work as blueprints that model how a physical asset could be created (Wilson et al., 2024) and as a training platform for operators and engineers that provides real-time feedback (Chen et al., 2020). Engineers use digital twins to combine historical data and real-time data to develop predictive analytics that monitor critical quality attributes, anticipate process outcomes and deviations, and develop proactive control strategies to automate quality assurance (Sokolov, 2020). Moreover, digital twins can simulate scenarios, predict product impacts, and develop response plans.

#### 4.2.2 In silico process design

In silico process design refers to the design of a process or unit operation based on the information available before the start of the process/operation. It is used to define an optimal operation strategy and designated set points and ranges (Sokolov, 2020), augmenting or replacing experimentation or studies. This capability involves experts in manufacturing processes and modeling to ensure the trustworthiness of the existing data and its ability to properly qualify the models used in process design. Advancements in interoperability and digital twin capabilities would allow integration of real-time data and PAT in the beginning of process design, where process development scientists would be able to simulate processes based on desired critical process parameters, CQAs of the end product, and facility capabilities (Rajamanickam et al., 2021). This would also support the scaling up of drug production from bench scale to commercial scale.

#### 4.2.3 Process diagnostic models

Process diagnostic models are tools that use data to identify static or dynamic problems and recommend possible solutions for specific business or operational processes. Models used in biopharmaceutical quality by design (QbD) approaches include statistical or chemometric models (which use design of experimental approaches with standard statistical equations), mechanistic models (which use complex, numerous, interdependent equations to represent known or hypothesized mechanisms), and hybrid models (a combination of AI/ML and mechanistic models) (Rajamanickam et al., 2021). These models can predict product quality (Zhou et al., 2022) and potential issues at different stages before they occur or diagnose the causal factors of events after they occur. They have applications for risk management, predictive maintenance (e.g., equipment recalibration, part replacement), and identification of control parameters and key performance indicators (KPIs) to help reduce downtime and batch failures. Additionally, these models can be used to analyze the product value chain (i.e., suppliers, manufacturing, packaging, product distribution) to identify bottlenecks and points of failure for correction.

#### 4.2.4 Data-driven decision systems

Data-driven decision systems involve automated electronic capture and analysis of data to inform and implement decisions and accomplish targeted outcomes. These systems provide the information that manufacturers need to be able to make objective data-based decisions, reduce uncertainty, and define and implement control strategies that optimize operations. They also can act as digital assistants to facilitate real-time monitoring and release, and automatically notify operators and supervisors of deviations and potential corrections or mitigations. This proactive approach ensures that corrective actions are taken in real time.

### 4.3 Secure shared datasets

Secure shared datasets are datasets that are created, curated, and shared to realize value while protecting the data from unauthorized access or misuse. These datasets require proper structure and secure platforms to ensure the data will be usable in modeling applications across users’ organizations while maintaining privacy and intellectual property. Sharing data within an enterprise or organization or between different enterprises facilitates collaboration, which can promote innovation and efficiency. Data sharing, however, presents risks; preventing unauthorized access and data misuse is particularly important in industries like biomanufacturing that are related to the health and safety of people. This capability can be difficult to implement because it requires access to privacy-preserving infrastructure, willingness and ability to share data, and high-quality data.

#### 4.3.1 Communal data

Communal data refers to collections of findable, accessible, interoperable, and reusable (FAIR) data created by and for a community that is committed to communal access. By leveraging shared and communally created datasets, biopharmaceutical companies can enhance the variety and comprehensiveness of data for collaboratively developing and training AI models. Additionally, communal raw material and consumables attribute and performance data aids identification of functionally equivalent alternatives. Privacy-preserving approaches and secure shared platforms used in combination with communal data mitigate risks while enabling use of a company’s proprietary data to create business value. For instance, communal data enables precompetitive collaboration for creation and innovation; subsequent “transfer learning” accelerates realization of proprietary applications for business value creation.

#### 4.3.2 Privacy-preserving computing

Privacy-preserving computing is an approach using encrypted, opaque, or distributed data or algorithms that enable data analytics while protecting the privacy and security of the data (National Science and Technology Council, 2023). Types of privacy-preserving computing include homomorphic encryption (computation with encrypted data), multi-party computation (jointly computing a function over private data without disclosing data), differential privacy (addition of statistical noise for obfuscation), and federated learning (central model trained with distributed models). End-to-end encryption and robust validation processes ensure that data remains confidential throughout its entire lifecycle. This level of security is crucial for maintaining the integrity of proprietary information and managing intellectual property rights while facilitating collaboration across organizations.

#### 4.3.3 Secure data linkage

Secure data linkage is the practice of combining data from multiple sources associated with the same entity while meeting privacy and confidentiality requirements (e.g., by using a blockchain). In biopharmaceutical manufacturing, secure data linkage enables lot-level traceability and identification of shared material lots between production batches. Similarly, a manufacturer may apply secure data linkage by tagging and serializing each dosage for its traceability and to differentiate authentic products from counterfeits. Sysrev is an example of a platform developed based on FAIR principles that facilitates data curation and evidence reviews/meta-analyses of the data (Bozada et al., 2021).

### 4.4 Interoperability

Interoperability is the ability of different information systems, devices, and applications to access, exchange, integrate, and cooperatively use data, both within a single plant and across sites/companies. The concept of interoperability can be described and assessed using the Levels of Conceptual Interoperability Model (LCIM), which classifies interoperability requirements into seven levels, including: None (L0), Technical (L1), Syntactic (L2), Semantic (L3), Pragmatic (L4), Dynamic (L5), and Conceptual (L6) (Wang et al., 2009). The LCIM framework is used as a maturity model; when higher levels are reached, the lower levels must have been satisfied. At lower levels of interoperability (L2), technical connections exist and agreed protocols exchange the right data, but the meaning of the data is not established. At middle levels (L4), systems are aware of the specific context and meaning of the data exchanged but are unaware of the larger use contexts. At the highest level (L6), systems are fully aware of each other’s information, processes, contexts, and modelling assumptions and how they change with time. Recognizing the needs of the different levels shifts the focus of the organization from basic integrability up to full interoperability, which is needed for effective digital twins. The advent of the Internet of Things (IoT) led to the need for ensuring secure and efficient interoperability across IoT devices; this is especially significant for industrial IoT (IIoT), where a lack of interoperability can cost companies billions of dollars (Wilson et al., 2024). Interoperability is highly dependent on having proper data structure and management practices, and its implementation is often affected by factors outside of an organization’s control, such as vertical integration into equipment or software companies’ proprietary systems that limit what types of new operations can be added to manufacturing processes. Because software companies lack incentive to support connections to their competitors, manufacturing organizations cannot overcome that limitation without switching to open-source solutions.

#### 4.4.1 Plug-and-play systems

Plug-and-play systems are technologies that allow a user to connect a smart digital device to a computerized system and use it immediately without manual intervention. These systems work by tagging devices from any brand or provider based on what they do and the types of data they create, and then automatically retrieving and implementing software to map them into the system—a type of self-reconfiguration (Soldatos, 2024). This capability makes devices easy to integrate into control strategies, including model predictive control. Plug-and-play functionality promotes flexibility in system design, enabling rapid equipment changeover, reconfiguration, and portability.

#### 4.4.2 Real-time data connectivity

Real-time data connectivity describes systems that enable connectivity over many data sources, applications, and servers for immediate access to data as it is generated. This capability focuses on planning and automating systems to capture, store, synthesize, and deliver data back to individual unit operations or instruments with very little latency. Platforms that enable seamless, real-time communication between models and equipment facilitate automation by reducing the need for manual interaction. They also aid technology transfers by ensuring data is captured at the receiving site and can be monitored in real time at the sending site. Digital twin models have a high demand for real-time connectivity from sensors and devices to ensure the digital replica reflects the current real state of the system (Acharya et al., 2024).

#### 4.4.3 Advanced sensors

Advanced sensors are soft sensors and other monitoring systems that incorporate models to extrapolate beyond the dataset they were informed on, increasing control and consistency of manufacturing processes across different facilities and scales (Nikita et al., 2023). These sensor systems deconvolute multivariate signals to separate phenomena within a broader trend, thus increasing mechanistic understanding of the system. These sensors enable real-time release testing and adaptive control by capturing multivariate process condition data—such as temperature, pressure, and chemical composition of individual components in mixtures—to monitor CQAs, alert when deviations are present, and enable rapid preemptive or reactive response. This capability saves time and money in raw material testing, process monitoring, and product CQA testing while reducing losses from failures.

### 4.5 Industry guidance

Industry guidance is composed of the guidelines and standards that embody current thinking in biomanufacturing to ensure compliance, consistency, and best practices within the industry. Guidance regarding the use of big data within biopharmaceutical manufacturing streamlines operations and practices, reducing the cost of investment, perceived risks, and overall barriers for company adoption of these technologies. Guidance may come in the form of regulations set by government agencies or best practices established by the leading companies within the industry. Best practices for workforce, such as training or talent management, help influence academic offerings and graduate competencies. Communal business cases help influence executive management to collaborate with competitors to achieve common benefit. All capabilities rely on this guidance, which is in turn informed by current practices and advancements in the industry. Without these resources, it is hard to establish a business case for new capabilities and innovations because organizations cannot establish a high level of confidence that they will lead to a quick return on investment through increased product registrations.

#### 4.5.1 Regulatory

Regulations refer to the rules and recommendations established by governmental or authoritative bodies to direct activities under their jurisdiction. Regulatory bodies publish guidance on various topics to help manufacturers adhere to regulations (Pharmaceutical Quality System, 2008; ISO 23247-1:2021, 2021; Luo and Zhang, 2023). FDA guidance applicable to biopharmaceutical manufacturing includes biologics guidance documents (Center for Biologics Evaluation and Research, 2025), guidance on the use of AI and ML in software that functions as a medical device (Center for Devices and Radiological Health, 2024), and guidance related to data submissions, such as the electronic Common Technical Document (eCTD) (U.S. Food and Drug Administration, 2024b) and Data Standards Catalog (U.S. Food and Drug Administration, 2025).

#### 4.5.2 Innovation business case

The business case for big data capabilities is a description of an opportunity and recommended action that is used to convince decision-makers to invest time, money, and resources on an innovation. The recommended action (in this case, implementing a big data capability) is based on applied insight from use cases aligned with a company’s business strategy, duration and magnitude of investment, risk and cost-benefit analyses, and return on investment (ROI) (Mikalef et al., 2018). For example, using AI’s visualization and predictive capabilities for inspection and automation is a major innovation that would support autonomous operations (Towards Resilient Manufacturing Ecosystems Through Artificial Intelligence - Symposium Report, 2022). The innovation business cases for many big data capabilities are largely shared and could be created openly together, as the business contexts and workflows are common within the community and large amounts of data are already shared by transactions between interacting enterprises. These shared business cases would show both the individual company benefits and the community benefits gained from collaborative development of specific capabilities, thus increasing communal partnerships and promoting alignment in the entire industry.

#### 4.5.3 Industry-tailored IT infrastructure

Biopharmaceutical IT infrastructure comprises best practice frameworks for delivering services that support an organization’s business processes (Fink and Neumann, 2007). Biopharmaceutical companies hire IT professionals and consultants to leverage their understanding of IT industry tools and apply it to the company’s operating processes and environments, helping the company successfully implement new IT tools and processes. IT professionals working in a biopharmaceutical manufacturing company can communicate with both infrastructure vendors and company management to bridge understanding gaps and reach mutually satisfactory solutions. When a company’s IT professionals promote strong partnerships between the IT industry and the biopharmaceutical manufacturing industry, companies are able to innovate and keep up with IT industry standards, leading to better, more applicable, and more biopharmaceutical industry-specific IT infrastructure capabilities (Fink and Neumann, 2007).

#### 4.5.4 Workforce development

Workforce development in the biopharmaceutical sector refers to the capability of a biopharmaceutical manufacturer to ensure its current workers are up-to-date on their skills and knowledge and that the company can hire and quickly train new workers (Conner et al., 2014). In particular, companies need to hire and train new workers with IT and data science skills who can then help train existing workers to broaden IT capabilities; current workers then help train incoming workers on the biopharmaceutical manufacturing skills they need to know, such as materials management and process design, transfer, and GMP execution in manufacturing (Conner et al., 2014). Benefits to individual companies drive their workforce development efforts; they will hire and upskill workers based on whether they have new technology or capabilities that require workers with the skills to leverage those capabilities (Towards Resilient Manufacturing Ecosystems Through Artificial Intelligence - Symposium Report, 2022).

### 4.6 Capability synergies

Each big data capability is linked to and relies on other capabilities; as a result, biopharmaceutical manufacturing companies cannot choose just one or two to integrate. For example, models cannot operate effectively without proper data structures, and shared datasets depend on standard privacy-preserving methods to ensure that the data being shared does not become compromised. These deep interdependencies enable the precision, scalability, and assurance of quality needed as the industry continues to grow; they are outlined in Table 1 below.

**TABLE 1:** Table of capability synergies. This table illustrates interdependencies between capabilities. The content in each data field of the table demonstrates how the capability in the column is influenced by the capability in the row heading.

Influencing capability	Data structure and management; A method of organizing and storing data within a computer system in a structured format to enable its future access, retrieval, and use	Modeling and simulation; Creation and use of virtual representations of a real-world system using software and hardware to emulate and evaluate complex systems	Secure shared datasets; Datasets that are created, curated, and shared to realize value while protecting the data from unauthorized access or misuse	Interoperability; The ability of different information systems, devices, and applications to access, exchange, integrate, and cooperatively use data	Industry guidance; Guidelines or standards that embody current thinking to help ensure compliance, consistency, and best practices within a particular industry
Data structure and management	​	Complex cross-enterprise data-driven models require standardized data structures (such as those produced by standards organizations or regulators)	Shared datasets must be structured so the varied parties can understand and use the data while ensuring privacy protections	Transmission and ingestion of data from and to diverse systems requires standardized data structures to ensure efficient and accurate use	Reliable and usable standards require proven existing extensible data structure and management tools for their implementation
Modeling and simulation	Technical details of data organization and storage are influenced by the use contexts (e.g., for low latency, highly featured digital twins)	​	The nature and quantity of data curated and shared depend upon the use contexts (e.g., patient observational data used for investigation models)	PAT/process analytical chemistry (PAC) device interoperability requirements are defined by model predictive control strategy	Regulatory agency guidance and the incoming workforce needs are influenced by industry trends for modeling and requirements for privacy preserving computing
Secure shared datasets	Data structure and management is influenced by user access privileges, needs for contextualization of communal data, and privacy preserving computing approaches	Models can be more predictive when trained with larger data collections representing the volume and variety of the community	​	Interoperability standards are influenced by IoT approaches to collect communal data to enable equipment predictive maintenance algorithms	Needs for industry guidance for privacy preserving computing are driven by industry concerns for privacy and needs for combining data collections for prediction accuracy
Interoperability	Data schema to encapsulate and transmit data are influenced by the dramatically expanding capabilities and data volume produced by emerging advanced sensors	Model temporal and spatial accuracy is determined by the latency and volume of data transmission from field devices	Development of communal supply chain resilience models relies on privacy and interoperability standards for transmission and contextualization of proprietary data	​	Standards for device and system interoperability are challenged by the growth of advanced sensors and proprietary data transmission approaches
Industry guidance	Data management and security approaches are designed and modified to ensure continuing compliance with approved standards and regulatory guidance	Digital twin models benefit from industry guidance to ensure scalability and interoperability with the spectrum of devices and systems employed	Risks associated with contribution to communal data collections and benefits from their use are highly influenced by standards for their curation and use	Absent standards and other guidance for interoperability, data transmission, structure, and contextualization defaults to proprietary licensed software approaches	​

Synergies between capabilities are demonstrated prominently in two current efforts from the National Institute of Standards and Technology (NIST). NIST created the Industrial Ontologies Foundry (IOF) as a mechanism to develop ontologies that promote data structure harmonization and interoperability capabilities across all manufacturing sectors, building on a core Basic Formal Ontology (BFO) (Kulvatunyou et al., 2022). Interoperable ontologies would enable more information sharing between companies to develop secure, shared datasets that advance industry science and demonstrate the business case for implementing more advanced big data capabilities. Digital twins are another example of many synergies existing between capabilities; they are complex systems that depend on adequate access to real-time data to be able to predict outcomes. NIST is leading the Digital Twins for Advanced Manufacturing project, an effort to develop standards for digital twins in manufacturing (National Institute of Standards and Technology, 2025). The technical advice provided through this project would help inform digital twin requirements for interoperability, model development, maintenance, analysis of results, and deployment across different lifecycle stages. NIST is also developing a Digital Twin Testbed through the project to support digital twin development within NIST.

## 5 Discussion and conclusion

The big data capabilities discussed in this paper intend to promote and support the entire biopharmaceutical manufacturing industry, from academic research and small companies to commercial manufacturing at all scales. Our framework is derived from extensive cross-enterprise subject-matter expert advice and uses the RBV perspective to identify resources and capabilities prioritized to address existing challenges and realize benefit. Conceptual alignment with the Horizon Europe analysis (Cavanillas et al., 2016) provides confidence of synergy with ongoing advances for other industry sectors. Similarly, alignment with the RBV strategic management framework (Grant, 1991) provides confidence for the business-value realization demonstrated for other industry sectors (Bharadwaj, 2000; Ravichandran and Lertwongsatien, 2005; Lin and Bush, 2010; Lu and Ramamurthy, 2011; Mikalef et al., 2018; Sabharwal and Miah, 2021) and aspects of biopharmaceutical therapy development (Lou and Wu, 2021).

It also conveys a future state that depends on overcoming adoption and implementation barriers for big data capabilities. In general, the biopharmaceutical industry resists implementation of “new technologies” owing to the ever-present high risk in new product development resulting from therapy complexity and the need for regulatory licensure. Some of the challenges associated with adoption of big data capabilities within a single biopharmaceutical manufacturing firm have been described (Grishikashvili, 2019) and include: technical (existing infrastructure, system integration, access management, *etc.*), managerial processes (culture transformation, alignment on goals, implementation strategy, *etc.*), and people (skills, mind-set). In general, big data initiatives (and associated capabilities) should be developed as dynamic capabilities and orchestrated by organizational management to achieve intended outcomes. In some cases, the need for regulators to accept and approve these new methods, and the need for a workforce with relevant knowledge and skills in both big data analytics and biopharmaceutical manufacturing are additional challenges.

Developing big data capabilities communally involves additional challenges which depend on the capability context. In the case of modeling and simulation, cross-industry modeling workshops have been sponsored by the “Recovery of Biological Products” and published recommendations for individual company advancements (Babi et al., 2022; Wittkopp et al., 2024). Development of reliable cross-industry standards for more complicated models like digital twins generally involves standards organizations (Ferko et al., 2023). Standards for implementing newer, more advanced modeling capabilities or manufacturing processes may not exist, causing companies to avoid potential risk associated with their adoption. Regulators, however, are moved by the current state of the art for the industry, frequently seeking input on new or revised standards that reflect current practices and advances.

In the case of developing communal data resources, companies are often unwilling to share their data to help build larger, shared datasets because of the desire to protect their IP. Similarly, equipment and software vendors have little incentive to encourage interoperability between their products and products from other vendors because they seek to sell their proprietary portfolio of interoperable equipment. There is, however, significant growth in the availability of open-source software and data repositories which offers pressure to change for the benefit of the community.

These challenges are formidable, but recent accomplishments demonstrate they can be overcome. A notable example is the MELLODDY Consortium, where ten pharmaceutical partners (Amgen, Astellas, AstraZeneca, Bayer, Boehringer Ingelheim, GSK, Janssen, Merck KGaA, Novartis, and Servier) shared decades’ worth of data through a private and secured computing platform to develop a shared quantitative structure-activity relationship model (QSAR) model—a model that uses the chemical structure of potential drug compounds to predict how they will function. Because it was informed on a large amount of communal data (more than 2.6 billion confidential experimental activity data points documenting more than 21 million physical small molecules), the MELLODDY QSAR model performed far better than all the individual QSAR models that the individual partners had developed using only their own data (Heyndrickx et al., 2024).

As this example shows, operationalizing the big data capabilities outlined in this paper benefits everyone in the community and individual organizational commitment can be achieved when the business case is persuasive. Companies that do not adopt these capabilities are competitively disadvantaged from realizing the full benefits of their big data in the biopharmaceutical manufacturing marketplace. NIIMBL, as one of the Manufacturing USA institutes, is a pre-competitive public/private partnership which includes academic, small company, industry, and government members to develop resources and capabilities to address industry needs and enables all members to realize benefit as they build their own unique strategies on common foundations. As such, NIIMBL is uniquely equipped to work with others in the community to grow development and adoption of big data resources and capabilities resulting from the Big Data Program and actively encourages participation. Together, the entire industry can accelerate innovation and unlock the full potential of big data for biopharmaceutical manufacturing.
