# Supplementary material for: Industry-defined opportunities for advancing big data capabilities in biopharmaceutical manufacturing
Source: Front Bioeng Biotechnol. 2026 Sep 4;14:1886721. doi: 10.3389/fbioe.2026.1886721 (PMC13586689; doi:10.3389/fbioe.2026.1886721)
Supplement: Supplementary file 1 [file Supplementaryfile1.docx]

Industry-Defined Opportunities for Advancing Big Data Capabilities in Biopharmaceutical Manufacturing: Supplemental Materials

# 1 March 2023 Workshop Process and Format

The NIIMBL Big Data Program held a workshop in March 2023 to gather input from NIIMBL members and other biopharmaceutical manufacturing industry stakeholders on opportunities for the use of big data to address challenges in biopharmaceutical manufacturing. These opportunities would then inform the future mission, vision, and technical focus of the work done in the Big Data Program. The Program worked with Nexight Group, a technical and management consulting company, to facilitate the workshop and work with the Program to develop its strategy as outlined in this paper and in another one currently in progress.

Workshop participants used XLeap to brainstorm and share big data capabilities related to each of the six biopharmaceutical manufacturing lifecycle stages. Figure S1 shows a sample of the input on capabilities specific to Data Transformation within the biomanufacturing lifecycle stage Process Design. Participants were asked to put their initial inputs into the “Unsorted” folder (seen first in the list on the left-hand side of Figure S1), after which they were sorted into one of the other five folders. Those folders were named after the previous names for the data value chain, which were tied to the Big Data Program’s workstreams (Data Creation, Data Transformation, Data Storage, Prediction from Data, Control from Data). They were renamed after the workshop to reflect the steps in the more widely used data value chain: Data Acquisition, Data Analysis, Data Curation, Data Storage, and Data Usage. It should be noted that these replacements are not directly one-to-one.

**Figure S1: XLeap Workshop Participant Input on Big Data Capabilities.** This is a snapshot of input from participants answering the question, “What Big Data capabilities related to Process Design should the biomanufacturing community advance to transform biopharmaceutical manufacturing?” Each idea is separated into one row with a number assigned by XLeap based on the order the input was received into the software across all questions. The results of the dot voting exercise (represented by the green circles with the number in them to the right of each numbered item) indicate which capabilities would be most impactful.

After the ideas were sorted into folders, workshop participants used dot voting to indicate which of the big data capabilities provided during the brainstorming session would be most impactful to the biopharmaceutical manufacturing community. NIIMBL members, non-NIIMBL members, and federal agency representatives were given different colored dots to compare the highest priorities of each of the three stakeholder groups. Voting was blind to collect a more objective representation of priorities; participants were shown the collective voting results at the end of the voting period. This voting exercise is displayed in Figure S2 below.

**Figure S2: XLeap Workshop Participant Voting Across Stakeholder Groups.** This is a screenshot of a separate interface in XLeap where facilitators combined all capabilities received for different stages of the biopharmaceutical manufacturing process (the ideas for Process Design are in the callout box featured), tagged with the area of the data value chain that each capability corresponds to. The data value chain tagging was completed in a separate session by a subset of the workshop participants. This displays the results of a voting session where participants were asked to use the color of dot corresponding to their stakeholder type to display the different aggregation of votes across stakeholder groups.

In the evening following the first day of the workshop, the facilitators took the capabilities and input them into Trello boards for an exercise the following day where participants would be sorting the capabilities based on when they would be able to be commercially implemented. One Trello board was created for each step of the biopharmaceutical manufacturing process, as well as one separate board for the highest priority capabilities. Each board included five categories: “Unsorted Capabilities” (where all capabilities were put to begin with), “Near Term” (indicating capabilities that could reach commercial implementation in 0–2 years), “Mid-Term” (indicating commercial implementation in 3–5 years), “Long-Term” (indicating commercial implementation in 6–9 years), and “10+ Years” (indicating commercial implementation in 10 years or more). Each capability was written on an individual Trello card that could be dragged and dropped on the board. The cards included the step of the biopharmaceutical manufacturing lifecycle that the capability applies to and a number in parentheses indicating the number of votes the capability received during the prioritization exercise in the workshop.

On the second day of the workshop, the facilitators led participants in an activity to sort the capabilities into the different timelines (which was accomplished by the facilitator dragging the cards based on participant consensus on where the capability on the card belonged). Participants were also asked to determine the current biomanufacturing readiness level (BRL) for each capability (designated as either Phase 1: Concept Development, Phase 2: Concept Demonstration, and Phase 3: Concept Realization). They were also asked to indicate which Big Data Program workstream(s) the capability best fits with. The BRLs and workstreams were tagged on cards using Trello labels. The workstream names were renamed following the workshop as described earlier. An example of a completed Trello board is included in Figure S3 below.

Figure S3: Trello Board with Big Data Capability Timelines. This is a screenshot of the Trello board of priority capabilities, showing where the capabilities were sorted in the timeline of commercial implementation. The step of the biopharmaceutical manufacturing lifecycle that the capability matches with is written on the beginning of the card and the number of votes that the capability received during the prioritization exercise is indicated by the number in the parentheses following the lifecycle step (e.g., “PROCESS DESIGN: (13)”) Capability cards are tagged with labels indicating the BRL of the capability (the tags beginning with “Phase”) and the Big Data Program workstream(s) they best fit with.

# 2 June 2023 National Meeting Process and Format

Following the workshop, the Big Data Program leads worked with the Nexight Group facilitators to begin synthesizing participant inputs based on the outcomes of the workshop discussion. The Big Data Program began developing its strategy for the next 5–10 years based on these synthesized participant inputs at the March 2023 workshop, which was shared for feedback and additional facilitated discussion at NIIMBL’s National Meeting in June 2023. Figure S4 below contains the program’s mission and vision statement as well as descriptions of the key big data capability themes and how they benefit biomanufacturing business units. Note that the key capabilities are tagged by the business units they benefit and the workstreams they fall under.

Figure S4: The NIIMBL-Led Big Data Program Strategy. This is a screen capture of a slide used during the NIIMBL National Meeting to present the mission and vision of the Big Data Program to NIIMBL members. It includes three areas of focus: the key capability themes (which went through additional reorganization following the National Meeting), business unit benefits (“business unit” was changed to “biopharmaceutical manufacturing lifecycle stage” in the paper), and the workstreams of the Big Data Program. The capability themes are tagged with both the business units that benefit most from the capabilities (noted with the colored dots to the right of the capability name) and the Big Data Program workstreams that are pursuing projects related to the capability theme (noted with the bracketed capital letter[s] next to the right of the capability name).

# 3 Sub-Capabilities and Their Effects on Business Segments

Table 1 below outlines the benefits for different business segments for each big data sub-capability organized by capability. Each capability is defined in a subheading row to the main table row, and each sub-capability included below the capability row is defined in the leftmost column.

Table 1: Sub-Capabilities and their Effects on Business Segments

| **Sub-Capability** | **Benefits for Process Design** | **Benefits for Tech Transfer** | **Benefits for Facilities and Engineering** | **Benefits for Supply Chain** | **Benefits for Manufacturing Operations** | **Benefits for Quality Operations** |
| --- | --- | --- | --- | --- | --- | --- |
| **Data Structure and Management:**  A method of organizing and storing data within a computer system in a structured format to enable its future access, retrieval, and use. | | | | | | |
| **Ontologies:** A formal semantic model that represents a set of concepts and their relationships within a domain. | Retrieve and contextually join data from disperse source systems to collect, analyze, and document data for specification of development history and design-space coverage. | Map sender and receiver data systems to a canonical model to enable cross-enterprise transfer of contextualized data for process, materials, equipment, analysis, and control. | Use common facility, equipment, parts, and materials data model for all plants to enable enterprise-level data resource management. | Use ontologies to holistically link different inventory management systems and provide insight for global procurement and inventory management. | Use ontologies to link elements within manufacturing execution system (MES) recipes to contextually related data such as equipment maintenance and material release. | Standard ontology/terminologies for knowledge management speeds queries into causes of failures and determining corrective actions. |
| **Data Schema:**  A formal and logical data model which describes data content, structure, format, and relationships for a bounded domain. | Data models for laboratory instruments support data transfer and interoperability with electronic lab notebook (ELN) system. | Process data is automatically packaged for digital transfer to a receiving system or placement in documents. | Packets of smart equipment performance data used for predictive maintenance with enterprise resource planning (ERP) system. | Supplier consumable data is placed in standard schema for transfer to and imported in customer system. | Automated trend monitoring receives and analyzes performance packets from MES system. | Automated quality management system (QMS) receives analytical results from Quality Control Laboratory Information Management System (QC LIMS) for real-time release. |
| **Holistic Data Integration:** Consolidating data from different sources with different formats into one easily accessible and interoperable system. | Automated integration of data from all systems and instruments used over the process lifecycle reduces data entry errors and improves awareness and reuse of prior results. | Use of a centralized repository for development and large-scale data which is accessible across sites reduces reliance on subject matter experts with historical knowledge and loss of data to silos. | Aggregation of all facility-level data (e.g., equipment, environment, water, power) in building management systems supports automated monitoring, prediction, and corrective action. | Consistent, accessible information about suppliers, customers, logistics providers, and other partners helps companies identify alternate suppliers and interchangeable products. | A system that aggregates data from equipment, sensors, and materials, and shows data linkages across the manufacturing lifecycle, improves process knowledge and product consistency. | Accessible, quality, comprehensive data (from large to small sets) lets operators and managers focus on decision-making. |
| **Knowledge Management:** A business process for creating, storing, using, and sharing knowledge and information within an organization. | Prior knowledge from similar processes guide platform process and control strategy specification. | Templates from prior technology transfers are continuously improved and reused. | Different manufacturing facilities within an enterprise align aspects of operations to facilitate multi-site regulatory compliance. | Supplier audit and qualification decisions are informed by prior experience and understanding of the current market alternatives. | Operators have access to historical data from a process, control strategy, and equipment to support training and investigations. | Specialists find and use contextually accurate knowledge artifacts to rapidly address deviation root-cause analysis and identify corrective actions. |
| **Modeling and Simulation:**  Creation and use of virtual representations of a real-world system using software and hardware to emulate and evaluate complex systems. | | | | | | |
| **Digital Twins:** A virtual replica of a real-world time-evolving object, process, or system which uses real-time data to simulate and predict real-world behavior. | Train model during entirety of process development to encode holistic design-space knowledge in future manufacturing support digital assistant. | Use model to explore potential deviation space associated with a specific facility implementation to forecast impacts and enable remediation. | Facility-level model holistically monitors environment and workflow to predict upcoming bottlenecks or disruptions and predict actions for avoidance. | Predict deviations in the product cold chain distribution arising from disruptive events to identify data-informed alternative routes for resilience. | Models informed by real-time process data support operators as digital assistants to predict and avoid process deviations before they occur. | Automated process validation and the ability to predict and respond to trends related to out-of-spec situations, shelf life, critical quality attributes (CQAs), etc. |
| **Process Diagnostic Models:** Tools that use data to identify static or dynamic problems and recommend possible solutions for specific business or operational processes. | Models automatically monitor analytical standard results from assays of in-process pools to identify changes in assay performance. | Use models to predict how well a process using one scale/facility/equipment will transfer to another. | Identify key performance indicator (KPI) and train models to predict when maintenance is needed to reduce costs and avoid failure of critical equipment. | Develop better inventory management systems that rely more on leading indicators and more inclusive use of all data sources. | Compare batch-to-batch trends to identify key drivers and factors for higher process yield. | Predict process steps where potential non-conformances could occur and preemptively act to reduce deviations. |
| **In-Silico Process Design:** Use of computer simulations with contextually appropriate data to develop processes by augmenting or replacing experimentation or studies. | Cross-modality models are used to determine process platform specifications to accelerate refinement of R&D process and technology transfer to manufacturing. | Accurately assess the impact of process changes to address facility fit remediations while ensuring process KPI and product CQA are unchanged. | Models with specific equipment and facility data are used to develop consistent standards to define facility capabilities. | Use of simulations to predict impact of alternate single-use system and consumable designs on process performance | Manufacturing process engineers assess process designs in development to enable early feedback and process refinement based on end-user needs. | Quality specialists collaborate with digital process design experts to assess the effectiveness of putative corrective actions to eliminate root-cause of deviations. |
| **Data-Driven Decision Systems:** Automated collection and analysis of data to inform and implement decisions and accomplish targeted outcomes. | A system assesses alignment of process design, analytical method, and control strategy with platform specifications. | A structured decision system compares process requirements with facility capabilities to determine facility fit and identify potential remediations. | Decision-based tool identifies equivalent equipment based on equipment design and performance specifications, and process performance requirements. | Use models to identify "functionally equivalent" consumables from alternate vendors to reduce single-supplier dependency. | Digital assistant automatically notifies operator and batch supervisor if anomaly is detected to inform and advise rapid responsive or corrective decision-making. | Decision/planning system tracks progress, acceptance, and completion of all Batch Production Record items to accomplish real-time release. |
| **Secure Shared Datasets:**  Datasets that are created, curated, and shared to realize value while protecting the data from unauthorized access or misuse. | | | | | | |
| **Communal Data:** Collections of findable, accessible, interoperable, and reusable (FAIR) data created by and for a community and committed to communal access storage. | Use shared and communally created datasets to enhance the variety and veracity of data for collaboratively developing and training AI models. | Collections of data inform process execution capability and provide increased awareness of CDMO offerings to originating organization. | Analysis of openly available equipment and system performance data informs user purchase and supplier improvement decisions. | Analysis of communal raw material and consumable attribute and performance data informs identification of "functionally equivalent" alternatives. | Communal libraries and software are used to extract data from multi-attribute methods (MAM) to enhance understanding and inform model building. | Open datasets of analytical method results from reference standard allows identification of magnitude and source of measurement variance. |
| **Secure Data Linkage:** Combining data from multiple sources associated with the same entity while ensuring privacy and confidentiality requirements are met. | Time-dependent analytical results from drug product formulation studies are linked through shared excipient, composition, product, and storage relationships. | Automation specifications for different equipment are linked to specific process recipe steps and the integrated control strategy to ensure accurate transfer. | Analyze private industry equipment performance data to identify communal KPI for model development and training to enable accurate predictive maintenance. | Securely share privileged material attribute and genealogy information about a raw material or consumable from supplier to customer. | Link time-evolving process analytical technology (PAT) measurement stored on historian with recipe-based human and automated actions to associate cause and effect. | Link process and quality data for sharing between CMOs and client institutions using tools for protection of confidential data to investigate batch failures. |
| **Privacy-Preserving Computing:** Approaches using encrypted, opaque, or distributed data or algorithms to protect data privacy while conducting computations for valuable purposes. | Collaborate with external experts to develop novel process steps and digital tools by combining data for greater variety while protecting proprietary data. | Rapidly conduct facility fit assessments with multiple CMO vendors to inform selection and contracting without disclosing user or vendor proprietary data. | Train predictive maintenance models using data from many manufacturers and user companies to enhance variety and volume without exposing proprietary data. | Train digital twin models with proprietary data to predict supply chain disruptions and identify responses to enhance system resilience for suppliers, logistic partners, and customers. | For cell therapy and personalized medicine, leverage private patient data to inform and validate manufacturing process changes to improve therapeutic outcomes. | Enhance understanding of impact of common process and product impurities on safety and efficacy measures while protecting patient privacy to improve linkage between product specifications and outcomes. |
| **Interoperability:**  The ability of different information systems, devices, and applications to access, exchange, integrate, and cooperatively use data. | | | | | | |
| **Plug-n-Play Systems:** Technology which allows a smart digital device to be connected to a computerized system and used immediately without manual intervention. | Process design data is rapidly and reliably collected from laboratory systems which were reconfigured to emulate a manufacturing site’s systems. | Plug-n-play functionality enables rapid equipment changeover and reconfiguration for early comparability and conformance runs. | Expand capability and flexibility of equipment systems by automated recognition and digital connection of as-needed devices and delay equipment obsolescence. | Use changeable testing equipment for identify and conduct specification testing of a portfolio of materials to reduce equipment purchase and maintenance costs. | Rapidly changeable robotic systems aid material sampling and delivery to point-of-use to maximize operator efficiency. | Smart devices record performance status, allowing equipment conformance to be confirmed as part of product release of deviation investigation. |
| **Advanced Sensors:** Soft sensors and other flexible monitoring systems that incorporate process knowledge to extrapolate beyond the dataset they were informed on. | Integrate sensors in the process that can accurately measure or predict CQA by analyzing complex collections of signals to inform control decisions for successful processing. | Expand the use of process analytical technology in multivariate sensors to improve process understanding and control in differing manufacturing facilities and scales. | Reduced need for physical intervention/presence in controlled/classified environments by relying on information from IoT-capable soft sensors. | Sensors enable track-and-trace ability throughout the entire product supply chain to identify and address disruptions, deviations, or adulteration. | Using smart sensors and PAT to have the right data at the right time to increase understanding, prediction accuracy, and control of the manufacturing processes. | Use of comprehensive, real-time data gathered by sensors help operators assess in real time whether product is in or out of specification. |
| **Real-Time Data Connectivity:** Systems which enable connectivity over many data sources, applications, and servers for immediate access to data as it is generated. | Provide interconnectivity between all monitoring instruments and controller to reduce latency in controller response and enable troubleshooting and model training. | Connectivity of data across scales and sites reduces manual effort required to align and analyze data between sending and receiving sites. | Capture, analyze, store, and feed data back to MES, system, or equipment controllers in real time to enhance fidelity of control and reduce human resource demands. | Reduce manual interactions needed for material management by developing robust, automated data collection and control systems. | Rapid and reliable changeover of model-based control modules utilizing different process measurements and control parameters. | Link data from multiple sources in context to create customizable datasets quickly for use in investigating deviations and identifying corrective actions. |
| **Industry Guidance:**  Guidelines or standards that embody current thinking to help ensure compliance, consistency, and best practices within a particular industry. | | | | | | |
| **Regulatory:** Rules and recommendations established by governmental or authoritative bodies to direct activities under their jurisdiction. | Collaboration with regulatory agencies for acceptance of advanced control methods and Industry 4.0 concepts enables their incorporation in process and factory design. | Collaboration with regulatory agencies to reduce risk associated with technology transfer, especially outside of U.S. jurisdiction. | Regulatory best practices guidance for use of robotics and other cutting-edge manufacturing systems to assist compliance with GMP and inspections. | Best practices for transaction recording with suppliers support compliance, variation management, forecasting, and interruption avoidance. | Guidelines for predictive modeling, AI/ML, digital twins, and other approaches for monitoring manufacturing operations enable development of risk management plans. | Industry standards for data requirements to support quality, efficacy, and safety information in regulatory submissions enhance communication with regulatory agencies. |
| **Industry-Tailored IT Infrastructure:** Best practice frameworks for delivering IT services to manage risk and deliver benefit while aligning with a company’s strategies, goals, and objectives. | Document common process design data lifecycle use cases with participant roles and ultimate consumers to inform data structure and management. | Develop a guideline for IT architecture at different scales to support data transfer; include small companies with few IT resources in the spectrum. | Define guidelines for data infrastructure to support the capture, storage, retrieval, and analysis of data including data security, cybersecurity, and data privacy needs. | Develop guidance on structure and features of cloud computing instances as markets for privately sharing supplier data with manufacturing customers. | Develop guidelines for aggregating data from operations, equipment, sensors, input materials, and environment using metadata for monitoring and reporting. | Guideline to harmonize data structure and storage promotes efficient collaboration among diverse organizations over the entire product lifecycle. |
| **Innovation Business Case:** A document which clearly describes an opportunity and recommended action which is used to convince decision-makers to invest time, money, and resources on an innovation. | Apply insight from use cases of data consumers and dataflows to make compelling case for data contextualization and ontology creation. | Develop and apply guidance for how a product innovator and contract manufacturer community can use privacy preserving computing to advance all members’ business needs. | Analyze current manufacturing site data with respect to zoning, labor, electricity, water, suppliers, transportation, etc. to identify attributes of desirable locations for manufacturing. | Describe the costs and benefits of using community supply chain data to address climate change issues and inform business decisions which benefit the company and the environment. | Develop compelling case for implementation of advanced automation and requisite big data systems by return on investment and other business value calculations. | Develop understanding of how the same data is used by different functional groups (e.g., process analytics data also used for quality review) to inform modernization plans. |
| **Workforce Development:** Best practices for setting strategy; conducting analysis; and developing, implementing, and monitoring action plans for talent management. | Companies possess a trained workforce who are prepared to implement Industry 4.0 aspects into modern facilities. | Workers are prepared to handle the intricacies of technology transfers, ensuring a smooth transition between companies/locations. | Workers are equipped to install, operate, and maintain advanced IT solutions in controlled GMP facility environments. | The company workforce can analyze complex global supply chain data to predict disruptions and model responses. | Workers can effectively leverage advanced manufacturing systems and manipulate/manage the data involved. | Companies possess a workforce educated and trained on ensuring data quality in a digital environment. |
